# Biomarkers of Volume Overload and Edema in Heart Failure With Reduced Ejection Fraction

**DOI:** 10.3389/fcvm.2022.910100

**Published:** 2022-06-17

**Authors:** Roxana Mihaela Chiorescu, Roxana-Daiana Lazar, Sándor-Botond Buksa, Mihaela Mocan, Dan Blendea

**Affiliations:** ^1^Department of Internal Medicine, “Iuliu Haţieganu” University of Medicine and Pharmacy, Cluj-Napoca, Romania; ^2^Department of Internal Medicine, Emergency Clinical County Hospital, Cluj-Napoca, Romania; ^3^Nicolae Stancioiu Heart Institute, Cluj-Napoca, Romania

**Keywords:** heart failure, volume overload, physiopathology, edema, biomarkers

## Abstract

From a pathogenetic point of view, heart failure (HF) is characterized by the activation of several neurohumoral pathways with a role in maintaining the cardiac output and the adequate perfusion pressure in target organs and tissues. Decreased cardiac output in HF with reduced ejection fraction causes activation of the sympathetic nervous system, the renin angiotensin aldosterone system, arginine-vasopressin system, natriuretic peptides, and endothelin, all of which cause water and salt retention in the body. As a result, patients will present clinically as the main symptoms: dyspnea and peripheral edema caused by fluid redistribution to the lungs and/or by fluid overload. By studying these pathophysiological mechanisms, biomarkers with a prognostic and therapeutic role in the management of edema were identified in patients with HF with low ejection fraction. This review aims to summarize the current data from the specialty literature of such biomarkers with a role in the pathogenesis of edema in HF with low ejection fraction. These biomarkers may be the basis for risk stratification and the development of new therapeutic means in the treatment of edema in these patients.

## Introduction

Cardiovascular diseases continue to represent a major health problem, being responsible for approximately one-third of global mortality and 45% of all deaths across European countries (1, 2). HFrEF plays a significant role in cardiovascular burden, and despite being extensively studied, its complex pathophysiology remains incompletely understood. Whatever the cause, symptomatic HFrEF is characterized by excessive accumulation of interstitial fluid due to sodium retention, with various implications in the quality of life, prognosis, and therapeutic management of these patients. Moreover, edema was reported to mark Stage C of HF (3, 4). Given the pathophysiology of edema, four theories have been suggested as contributing to extracellular fluid expansion, namely: a reduction in glomerular filtration rate (GFR), a stimulation of the renin-angiotensin-aldosterone pathway, activation of renal sympathetic nerve fibers and inhibition of natriuretic component (5).

Comprehending these mechanisms allows both the understanding of the HF clinic and a logical therapeutic approach, because the treatment aims to correct the excessive compensatory mechanisms, which themselves became the cause of perpetuation and aggravation of the patient's suffering.

Sodium and water renal retention is the first response mechanism that leads to edema formation and a redistribution of body fluids in HF. Additionally, adjustments in the venous abdominal reservoir and central cardiopulmonary vascular beds were also reported to contribute to clinical congestion, impacting right heart hemodynamics that can be used to track alterations in volume status (6). Throughout the years, various biomarkers have been proposed to guide the management of HF. However, there is less data on how biomarkers are linked to edema formation, and in which extend can be used for prognostic, clinical or therapeutical purposes.

## The Main Pathophysiological Mechanisms Involved in the Production of Edema in HFrEF

### Redistribution of the Cardiac Output

HFrEF is characterized by a decrease in heart rate because of various factors that act upon its main determinants (contractility, overload, post-pregnancy, frequency). The decrease in cardiac output in HF is perceived by the body similarly to the decrease in volume. A cascade of mechanisms gets activated and determines the renal retention of water and salt. These mechanisms consist mainly of the redistribution of the cardiac output, which causes changes in the renal hemodynamics and neuroendocrine stimulation. These two main mechanisms (redistribution of the cardiac output and neuroendocrine stimulation) are fundamental for the retention of water and salt in the body and cause edema (7). Also, in HF there is a redistribution of the cardiac output to the essential organs to the detriment of the others. Thus, the flow of the coronary circulation is maintained, the cerebral flow decreases a little and there is a significant drop in the cutaneous and renal circulation. The decrease in cardiac output and its redistribution causes a reduction in renal flow and as a result a decrease in glomerular filtration is seen. Hence, intrarenal blood redistribution occurs and juxtaglomerular nephrons with high concentration capacity will be irrigated preferentially. Normal tubular resorption will result in the formation of a reduced urinary output and in the retention of water and salt (8, 9).

The decrease in renal flow causes an increase in renin release and angiotensin formation, stimulating the secretion of adrenal aldosterone. As a result, the sodium-potassium tubular exchange increases with sodium retention. Decreased renal flow increases the secretion of antidiuretic hormone with water retention. The neuroendocrine mechanism and renal hemodynamic changes cause hydro-saline retention. As a result of the hydro-saline retention when under an increased venous and lymphatic capillary pressure, edema occurs, which in turn lead to the increase in the interstitial pressure with the compression of the small vessels and the increase in the rigidity of the arterial walls (10).

### Neuroendocrine Activity

**Figure 1:**
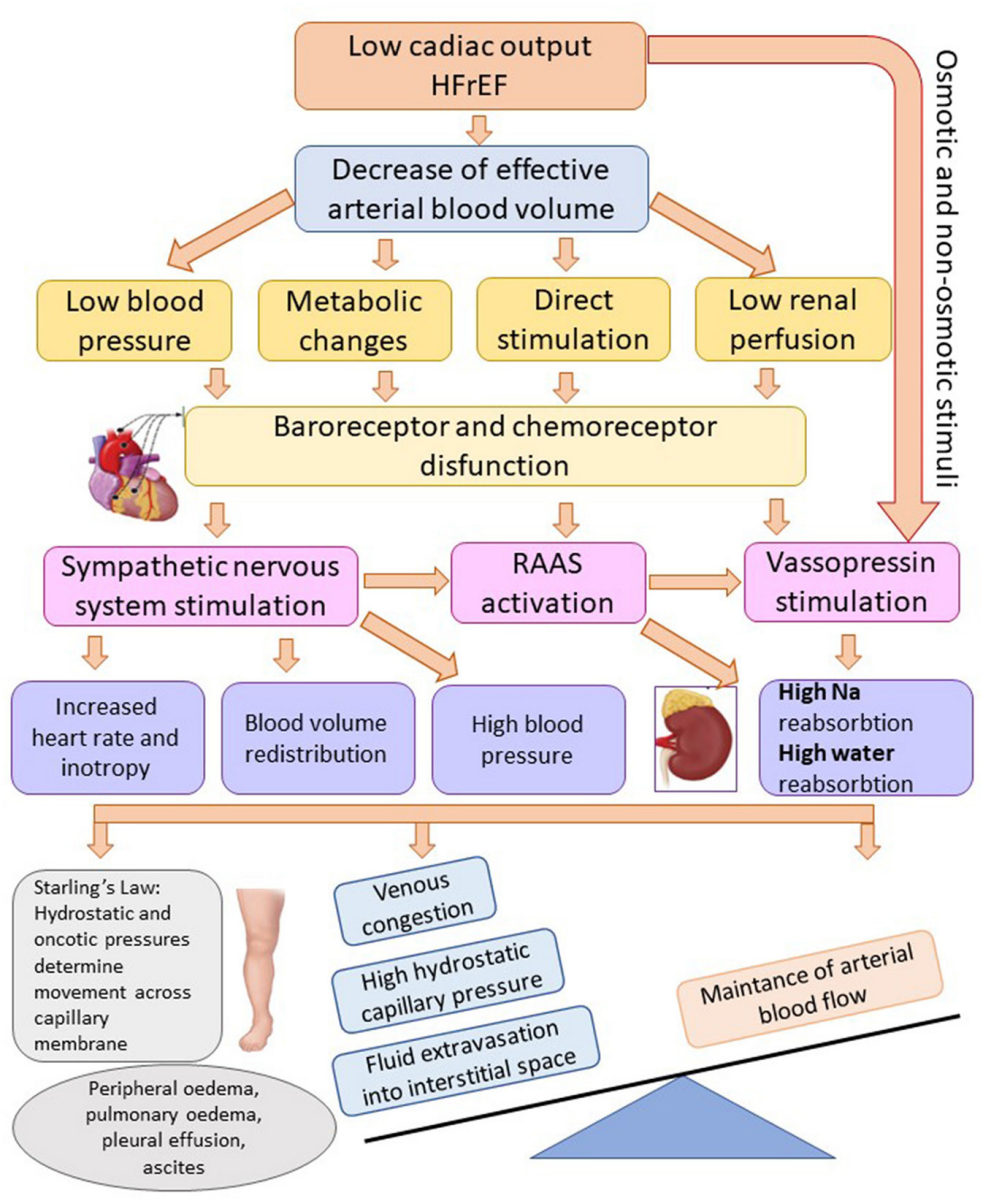
Mechanisms of fluid overload and edema in heart failure with reduced ejection fraction. Adapted after Adams et al. (8) (HFrEF, heart failure with reduced ejection fraction; Na, sodium; RAAS, renin-angiotensin-aldosterone system).

Triggering and coordinating the compensatory mechanisms at the central and peripheral level with the realization of an initially useful adaptive reaction, but which, after being ended, becomes pathological because of the activation of complex neuroendocrine mechanisms. Some of these are vasoconstrictors and others are vasodilators. Neuroendocrine activity causes not only hemodynamic adaptation, and hydro-saline retention, but also cardiac and vascular remodeling. The main vasoconstrictor mechanisms are: the sympathetic stimulation, RAAS, arginine-vasopressin system, endothelins, and the main vasodilatory mechanisms are: the natriuretic system, endothelial vasodilator factor, prostaglandins. The vasodilator systems have an antiproliferative and apoptosis-reducing action, while the vasoconstrictor ones trigger cardiac remodeling processes that involve the proliferation of muscle and nerve tissue and trigger apoptosis (11). Main pathophysiological mechanisms involved in the production of edema in HFrEF are synthetized in Figure 1.

- **The sympathetic stimulation** is triggered by the stimulation of vascular baroreceptors stimulated by tissue hypoxia. The sympathetic stimulation causes an increase in the release of norepinephrine from the postganglionic terminals and adrenaline from the adrenal medulla. The sympathetic stimulation determines tachycardia and increased contractility at the central level while at the peripheral level it causes vasoconstriction with redistribution of the cardiac output to the periphery, redistribution of the renal plasma flow to the juxtamedullary nephrons, and at the endocrine level the stimulation of renin secretion and other mediators (7).
- **The RAAS** is activated by: the decrease in the prefusion pressure of the afferent glomerular arteriole, decrease in the sodium concentration at the level of the dense macula, and by the sympathetic activation. Circulating renin converts liver angiotensinogen to angiotensin I (AT I), which is converted by the enzyme converting angiotensin to the active mediator - angiotensin II (AT II). AT II has a potent vasoconstricting actin, increases the release of catecholamines from the nerve endings and the adrenal medulla, and stimulates the release of aldosterone. Thus, the RAAS participates in the redistribution of the cardiac output, salt retention and it maintains the sympathetic stimulation (12).
- **The arginine-vasopressin system** is stimulated by osmotic receptors, baroreceptors or directly by AT II. The activation of this system contributes to vasoconstriction and water retention (13, 14).
- **The endothelin system** is stimulated by tissue hypoxia. It causes vasoconstriction, increases inotropism and has a proliferative effect on myocytes and fibroblasts (15).
- **Prostaglandins** are activated because of increased vasoconstrictor activity. Vasoconstrictor systems are responsible for blood flow redistribution. Thus, at the renal level, the increase in the Ang II concentrations and the renal ischemia determines the increase in the prostaglandins with local vasodilating effect (16, 17).
- **Natriuretic peptides (NPs)** have vasodilating and antiproliferative action. They are synthesized in the cells of the cardiovascular system:
  - Natriuretic peptide A (ANP) from the atrial and ventricular myocardium, the natural stimulus is atrial dilation.
  - Natriuretic B peptide (BNP) released by the ventricular myocardium in response to the increase in the filling pressure;
  - Endothelial cell peptide C under stimulation of parietal shear force caused by the blood flow.

Natriuretic peptides increase the excretion of sodium and water, have vasodilating action, inhibit the secretion of norepinephrine, renin and endothelin (18).

## The Diagnosis, Prognosis, Management and Even Risk Stratification of the Patients With HFrEF

The diagnosis, prognosis, management and even risk stratification of the patients with HFrEF was synthetized both in the newly published guidelines of AHA/ACC/HFSA in 2022 (19) and in ESC guidelines in 2021 (20).

The new classifications of HF based on left ventricular ejection fraction (LVEF) as recommended by the 2022 AHA/ACC/HFSA Guidelines are as follows:

- HFrEF corresponding to LVEF ≤ 40%.
- HF with improved EF (HFimpEF) corresponding to previous LVEF ≤ 40% and a follow-up measurement of LVEF >40%.
- HFmrEF corresponding to LVEF 41%−49% and evidence of spontaneous or provokable increased LV filling pressures (19).

Heart failure with preserved ejection fraction (HFpEF) corresponds to LVEF ≥50% and presents echocardiographic evidence of spontaneous or provokable increased LV filling pressures.

Under the new guidance, the risk for HF was stratified in stages. The first stage identifies the risk factors for HF early (stage A), and those at risk of HF. In addition, the guidelines will help provide treatment before structural changes or signs of decreased heart function occur (stage B). In other words, stage A is defined as “at risk for HF,” stage B as “pre-HF,” stage C as “symptomatic HF,” and stage D as “advanced HF” (19). In the ESC Guidelines, the recommended biomarkers for the positive diagnosis of HFrEF are NPs (BNP and NT-proBNP). In oncologic patients both Troponins and NPs were proved to be useful for the early detection of cardiac disfunction and follow-up during the chemotherapy (particularly anthracyclines or trastuzumab). Both guidelines clearly confirm the clinical utility of serum NPs (BNP or and NT-proBNP) for establishing disease and prognosis in chronic HF, regardless of etiology (20).

Clear recommendations of biomarkers use were introduced in the new guidance of 2022 AHA/ACC/HFSA. An entire section is dedicated to NPs and their use in emergency for the etiology of the dyspnoea and exclusion of HF, for risk stratification in chronic HF, to establish the prognosis at admission and at discharged after an episode of acute HF, and at last to stratify the patients with risk for developing HF. However, there are insufficient evidence to inform specific guideline recommendations related to NPs-guided therapy or serial measurement of BNP or NT-proBNP to reduce hospitalizations or mortality (19).

Persistence of pulmonary congestion after hospitalization is a negative prognostic factor in patients with decompensated acute HF. Quantification of pulmonary congestion can be done by quantifying the radiological score of congestion, using a systematic approach in six lung areas. Clinical studies have shown that this parameter better identifies the risk of readmission of these patients compared to BNP, physical assessment (NYHA class) and echocardiographic parameters (21). Patients with HFpEF develop exertional pulmonary congestion. Determining the degree of this can be done with the help of exercise pulmonary ultrasound by quantifying the B lines in patients with elevated BNP and echocardiographic parameters of diastolic dysfunction (22).

Unfortunately, the role of other biomarkers of myocardial fibrosis such as *ST2* or galectin-3 was not well established, and they were not introduced in clinical practice.

As for the management of HF, 2022 AHA/ACC/HFSA Guidelines included sodium-glucose cotransporter-2 inhibitors (SGLT2i) in the guidelines reflecting the availability of more proven treatment options for both HFpEF and HFrEF. The treatment of hypertension and atrial fibrillation has been updated, along with updates recommending avoidance of routine use of nitrates or phosphodiesterase-5 inhibitors. Moreover, the therapeutic options were classified in low-value, intermediate value and high-value treatments. Tafamidis for cardiac amyloidosis was the only low-value therapy identified. ARNi, ACEi, ARB, beta blocker, MRA, implantable cardioverter-defibrillator, and cardiac resynchronization therapy were classified as high value, while SGLT2i inhibitors and cardiac transplantation were classified as intermediate value therapies (19).

Patients with advanced HF should be addressed to a specialty team in order to increase life expectancy, comorbidities like iron deficiency, anemia, high blood pressure, sleep disorders, type 2 diabetes, atrial fibrillation, coronary artery disease, and cancer should receive special attention (19). Benefits of physical activity and increase in quality of life was observed after systematic rehabilitation programs in patients with HFrEF, but at the moment the biomarkers are not used for the surveillance after rehabilitation (20, 23).

Overall, the new definitions, classifications, and risk stratification in stages of HF are the first steps for us to achieve clarity and uniformity of care to timely diagnose and target appropriate guideline therapies.

## Biomarkers With Clinical Implications for Edema in HFrEF

The onset of neurohormonal mechanisms in HF has an adaptive role and succeeds in the first phase of compensated heart failure - stage B the control of the symptoms. If the stimulation of these neurohormonal mechanisms continues excessively, their ability to compensate for the pathophysiological changes in heart failure will be overcome and will lead to disease progression. Thus, prompt therapeutic intervention, at the right moment, on these neurohormonal mechanisms will prevent the progression of the disease (20).

### Biomarkers of Sympathetic Activation

During physiological conditions, cardiac activity is modulated by the sympathetic and parasympathetic nervous system *via* chronotropic, inotropic, dromotropic and lusitropic properties. Adding to this the balance in the peripheral resistance, we get cardiac output (24). Stress conditions are associated with an increased release of **catecholamines** [**CA**; **adrenalin** (**AD**) and **noradrenaline** (**NA**)] from brainstem catecholaminergic neurons, spinal cord efferent nerves and adrenal medulla. The functional consequence of the sympathetic overdrive, seen in chronic HF, is represented by a chronic elevation in both circulating levels of plasma CA and their urinary excretion (24, 25). Thus, the myocardium has to adapt to current cardiovascular demands (both energetic and metabolic) expressed by tachycardia, vasoconstriction and sodium and water retention, in order to maintain heart's function of cardiac pump. Nevertheless, these mechanisms have deleterious effects on cardiac structure and performance, leading not only to symptomatic HF, but also to HF decompensations. In addition, higher levels of plasma circulating NA seen from Stage B in HF, were strongly linked to a worsening of systolic dysfunction and a progression of interstitial fluid accumulation, marking the onset of edema formation at biochemical/molecular level (26–30).

Results of experimental studies have shown that the suppression of central sympathetic activity by renal denervation (ablation of renal nerves, also used as an alternative for the treatment of resistant hypertension in humans), had therapeutic effects on HF, reducing tissue edema (9, 27).

Furthermore, some studies revealed that renal denervation improved natriuretic and diuretic response, especially in conditions predisposing to acute volume expansion. Nevertheless, as the results from denervated HF patients were significantly different from the denervated control specimen (rats), the authors suggested that besides this procedure, other factors/biomarkers that contribute to the improvement in sodium retention during HF should be explored (8, 28).

**Catestatin (CTS)** and its precursor, **chromogranin A (CgA)**, are other biomarkers implicated in sympathetic activation. Several studies focused on the establishment of their physiological role in patients with HF. Being produced by nerve fibers and neuroendocrine tissues, they exert a downregulation of CA release, providing cardioprotective effects against excessive sympathochromaffin overactivation. CTS, in addition to its prevalent vasoreactivity, expresses cardiosupressive effects (negative inotropic and lusitropic actions) *via* β2- and α-adrenoceptors found on the myocardial cells surface (31). In other words, CTS's modulatory actions have a direct impact on cardiac cells intrinsic health.

Specifically, CgA demonstrated to be a powerful predictive marker of mortality and morbidity in HF subjects (28). It inhibits cardiac remodeling, reduce arterial blood pressure and decrease endothelial and vascular inflammation (24, 28, 29). The last two mentioned effects may suggest its involvement in body fluid homeostasis, thus an increased circulating plasma level of CgA observed in HF patients, would translate as a compensatory mechanism meant to limit acute/chronic fluid overload. However, further studies to evaluate its relationship with edema formation, are needed.

**Adrenomedullin** (**ADM**) represents a hormone secreted mainly by the gastrointestinal cells that exerts several physiological functions, among which the most relevant are vasodilation, anti-inflammation, tissue repair and organ protection (30, 32). ADM is normally found in healthy individuals' plasma in low concentrations (33). Its effects are believed to be related to the modulation of sympathetic nervous system during stress conditions. Moreover, ADM release was shown to be stimulated by fluid overload, in order to combat further vascular leakage, thus limiting congestion and maintaining the endothelial barrier function (34). Taking these into account, several studies revealed that higher levels of plasma ADM are markedly increased in HF, corelating with the aggravation of signs and symptoms of remaining congestion, and in consequence, with disease severity (30, 34, 35). In addition, a rise in the use of loop diuretics at discharge and greater risk of readmission due to decompensated HF, were predicted by increased levels of ADM. These results proved that ADM could be a useful marker to identify HF subjects at risk of rehospitalization due to suboptimal decongestion (34). Another study proposed that the stabilizing endothelial barrier effect, could be achieved by administrating 15 ng/kg/min of ADM, thus reducing pulmonary congestion without significant hemodynamic effects (36). Unfortunately, since ADM plasma levels are also very elevated in severe infections (especially in septic status), its use in HF patients must be carefully interpreted. From another point of view, ADM could also be used as a target therapy in HF. The ACCOST-HH Trial hypothesized that Adrecizumab (ADZ), a non-neutralizing anti-ADM antibody, binds to ADM, prolonging its half-life, reinforcing the fact that it is not an inhibiting antibody (37, 38). In theory, ADZ allows shifting of ADM from the interstitial space into the blood vessels (33, 38). Consequently, the bioavailability of ADM will increase and its beneficial effects will be enhanced. However, since an increased plasma level of biologically active ADM (bio-ADM), correlates with a high mortality risk, whilst its plasma level normalization, correlates with a decrease in mortality (39), it becomes a controversial subject of whether supplemental administration of ADM would really be beneficial among HF patients.

### Biomarkers of Renin–Angiotensin–Aldosterone System (RAAS) Activation

Renin-angiotensin-aldosterone system (RAAS) enhances and contributes to the built of the congestive syndrome seen in HF *via* its chronic activation. The pathophysiologic impact of the RAAS is mainly guided by AT II and aldosterone. The adverse cardiovascular events are similar to those observed in sustained sympathetic activation and excessive CA release, amongst which, vasoconstriction, and sodium and water retention, are included (8). Moreover, the congestive syndrome seen in HF associated with renal dysfunction, is usually difficult to evaluate and tends to a worsen HF. Nevertheless, signs and symptoms of excessive volume overload may be present even in patients with HF and preserved EF due to renal impairment (14), proving a close interrelation between the kidneys, through RAAS activation, and sympathetic nervous system (SNS) (26). This is also proved by, low circulating levels of AT I and II that were seen after radiofrequency renal denervation (9). The implementation of RAAS blockers in the last decades, has led to a significant reduction in the rates of hospitalization and mortality among HF patients (40).

#### Prorenin/Renin

With regard to the impact of renin in the congestive HF, Sullivan et al. (41) concluded that before the onset of Stage C HF, which is associated with fluid retention, experimental animals expressed higher levels of plasma renin activity. It is the first structure released from the juxtaglomerular apparatus in the RAAS cascade. Studies have shown that the progression of HF is held back due to a decrease in sodium and water reabsorption, amongst patients with normalized plasma renin activity. Ergo, despite the fact that the first two generations of direct renin inhibitors (DRI) failed to show valid clinical benefits, Aliskiren proved to delay progression of HF from stage B to stage C, as a result of significant reduction in the development of edema (41). Also, adding DRI to the standard HF therapy was shown to carry great potential benefits in HFrEF patients with elevated plasma renin activity (26).

#### Aldosterone

Aldosterone acts on sodium handling in the renal distal tubules and it is considered to be a key-hormone in the progression of HF, through its effects on the vessels, heart and brain (26, 42). Even though its production is stimulated by AT II, other hormones and mediators have been shown to act on its production and release. In that matter, angiotensin converting enzyme inhibitors (ACEi) proved to produce a transient drop in plasma levels of aldosterone (8). Regarding its potential benefits in combating edemas, Girerd et al. (40) reported that in patients hospitalized for severe diuretic resistance and concerning hypokalemia, intravenous mineralocorticoids antagonists (MRAs), such as canrenoate, attained decongestion and could be used as a rescue therapy. Furthermore, aldosterone blockade could be also achieved using aldosterone synthase inhibitors (ASIs) as an alternative to MRAs. The advantage of this novel approach lies within the fact that ASIs do not induce negative feedback and eventually increased cortisol (which in turn exert various side effects, increasing even cardiovascular risk *via* prolonged activation), as opposed to MRAs. Their effects were studied in patients with primary aldosteronism, displaying a reversible dose-dependent reduction in aldosterone concentrations, both plasmatic and urinary. Also, clinical tolerance and biological safety were comparable to those of eplerenone (43). However, these blockades are not always sufficient since there were described cases in which a rebound increase in circulating aldosterone was described when using MRA combined with ACE inhibitors (44). This phenomenon, called aldosterone breakthrough, is still to be cleared up.

Angiotensin and aldosterone physiologically increase the apical permeability of the collecting ducts to sodium, modulating the activity of epithelial sodium channels (ENaC) (45). Since these biomarkers show elevated plasma concentrations in HF, it was hypothesized that their chronic overdrive might generate a malfunction of ENaC. Zheng et al. (5) proved in an experimental study, that rats with HF expressed an abundance of protein subunits of ENaC in the cortex and medulla of the kidneys.

**AT II** is the major effector of RAAS (37). AT II plays an important role in the short-term maintenance of circulatory homeostasis, but its sustained production has maladaptive effects and leads to fibrosis of the heart, kidneys, and other organs, as well as to the worsening of neurohormonal activation by increasing the release of norepinephrine from the sympathetic nerve endings by stimulating the adrenal medulla for aldosterone release. Aldosterone supports short-term circulation by renal reabsorption of sodium in exchange for potassium, but in the long-term it has negative effects through hypertrophy and fibrosis at the myocardial and vascular level, decreasing vascular compliance and increasing the myocardial rigidity (8).

**Angiotensin converting enzyme 2** (**ACE2**) is a counter regulator of RAAS with the role of opposing the effects of AT II by transforming it into AT I (10), which has a protective role on the cardiovascular system (11). The value of ACE2 is increased in patients with HF and systolic dysfunction alongside an unfavorable evolution. Thus, ACE2 is a biomarker with a prognostic role for patients with HFrEF (12). New drugs that target ACE2 were shown to attenuate diastolic disfunction in HF subjects. SARS-CoV-2 virus uses host-receptors of ACE2 to attach and invade human cells and cause multi-organ lesions. Comorbidities such as HF and coronary artery disease express elevated levels of ACE2, thus leading to an increased susceptibility and disease severity in SARS-CoV-2 infection. Also, the binding of the virus to ACE2 receptors determines endothelial dysfunction through hyperinflammation, elevated plasma levels of prothrombin, and as a consequence the risk of arterial and venous thrombosis increases (46–48).

ACEi use in SARS-CoV-2 infected patients has been controverted, since various authors have raised concern about the fact that they will increase infection susceptibility due to raised expression of ACE2. Despite this, ARBs were shown to be superior to ACEi in this category of patients, and treatment continuation has been proposed. However, other authors claimed that ACEi can be in fact benefic against pulmonary lesions induced by the virus and should be introduced in the treatment strategy of COVID-19 patients (49).

Additionally, a meta-regression analyses shows that ARBs/ACEi use, correlates with a significant drop in mortality in COVID-19 patients, after sex, age, cardiovascular disease, high blood pressure and chronic kidney disease adjusting. Moreover, treatment management of COVID-19 patients from current guidelines also recommend continuation with these pharmacological agents in patients with cardiovascular comorbidities (46).

**The apelin**/**APJ** system also has a role in counter-regulating RAAS by antagonizing AT II activity (50). **Apelin** is a peptide encoded by the APLN gene, located on the long arm of the X chromosome. It is originally produced as a pre-pro-apelin consisting of 77 amino acids. Apelin is subsequently cleaved into smaller C-terminal fragments with biological activity (apelin-13,−16,−17,−19,−36) (51). Apelin-13 is the shortest fragment derived from pre-pro-apelin and it is the most potent peptide from all the fragments cleaved by angiotensin-converting enzyme from this 77-amino acid protein. At the cellular level, apelin exerts its effects by activating B-protein kinase pathways (Akt), Cε protein phosphatidyl-inositol-3 kinase (PI3K) and regulated extracellular signaling kinases (Erk), proteins known to play a role in preventing apoptosis and cell proliferation and migration. Apelin has complex functions in the body, being a potent inotropic, vasodilator and diuretic agent. The apelin/APJ system has many physiological actions on the hydric balance. The latter effect is produced by inhibition of the vasopressin secretion at the neuronal level and by actions on renal microcirculation and tubular function. During the evolution of HF, the plasma level of apelin decreases gradually after an initial rise in the early stage of the disease (52, 53). There is evidence that ACE2 is the enzyme which fragments apelin by removing the C-terminus, the main area through which it exerts its effect on the APJ receptor (51, 54). In patients with HF with a low ejection fraction and an unfavorable prognosis, apelin has reduced values, thus being a biomarker with a prognostic role (55, 56).

### Biomarkers of Arginine-Vasopressin System Activation

**Copeptin** represents a C-terminal subdivision of the precursor pre-pro-vasopressin, and similar to NT-proBNP, directly reflects arginine-vasopressin (AV) levels (which are difficult to measure and not used in clinical practice). Its secretion is stimulated by hyperosmolarity and exposure of the body to different stages of endogenous stress, as a result of AV system activation (57). The MOLITOR study (58) showed that high copeptin level at admission proved to be the best prognostic marker of rehospitalization and 90-day mortality rate, in conjunction with the worst prognosis at 3 months follow-up of patients with increased copeptin serum levels during the first 3 days of hospitalization. Also, its predictive value was much the same as NT-proBNP's in terms of all-cause-mortality, or even superior (59, 60). When combined with sodium, copeptin provided increased prognostic information for HF mortality and correlated with other markers of congestion, proving its involvement in pathophysiological mechanism of fluid retention (60).

### Biomarkers of Kinin–Kallikrein System Activation

In the distal nephrons, the kinin–kallikrein system (KKS) modulates water and sodium reabsorption by acting on the ENaC gating. Increased kallikrein urinary concentrations following administration of antihypertensive drugs displays KKS's interaction with other biologically active molecules such as prostaglandins and RAAS, as well as its involvement in their mechanism of action (61). Evidence suggests that bradykinin (BK) acutely repress ENaC activity in a reversible and dose-dependent manner (62). Moreover, BK was shown to generate a decrease in ENaC opening probability, thus blocking sodium and water reabsorption. Hence, in experimental studies on mice, augmented ENaC action with subsequent hypertension were observed following genetic ablation of BK receptors. Likewise, increased BK signaling revealed a powerful antihypertensive effect, proving its value in the biological natriuretic response and clinical additional benefits in decongestion (61).

Also, in spite of being a paradox given its name, angiotensin converting enzyme (ACE) seems to be a much more potent catalyst in cleaving kinins, than AT I in AT II (15). This became unquestionable when ACE inhibition was followed by a considerable increase in plasma concentration of BK. On the bright side, among BK's therapeutic effects, vasodilation and reduction of sympathetic overdrive were just “the tip of the iceberg” (8). Therefore, suspicions were raised on whether the clinical improvement seen in congestive HF patients should be attributable to the inhibition of AT II production, or much likely to the augmentation of BK.

Finally, other proteases are noteworthy due to their effect on the activation of ENaC. A recent study revealed that patients with HF and signs of clinical congestion presented significantly elevated urinary concentrations in plasmin, furin and prostasin, confirming their involvement in edema formation (63).

### Biomarkers of NPs Activation

Natriuretic peptides have long been used to assess the level of congestion in HF. Their plasma concentrations are increased as a response to volume and pressure overload in the myocardial walls. The diuretic and natriuretic effects of NPs as an attempt to reduce blood volume, is generated by antagonizing the actions of AT II on vascular walls (reducing peripheral vasculature resistance), aldosterone secretion, and sodium and water reabsorption (9, 64). Also, Bagshaw et al. (10) observed that NPs proved to be useful in assessing interstitial fluid overload, that eventually leads to acute decompensations of HF, associated by clinical exacerbation, hospitalization, and death. Herein, the use of these biomarkers could be translated into earlier therapeutical interventions and better clinical outcomes.

Nevertheless, a prospective design study containing 565 cases of sudden cardiac deaths (SCD) disclosed that even a subclinical volume and pressure overload triggers NT-proBNP and leads to an increased risk of SCD before the onset of clinical signs and symptoms of HF, also reflecting its prognostic value as a marker of myocardial injury and hemodynamic stress (65). Finally, from a statistic point of view, NT-proBNP's diagnostic accuracy for HF shows a specificity of 90%, a negative predictive value of 95%, and a positive predictive value of 85% (66).

Sacubitril/valsartan combination acts as first line blocker of angiotensin-neprilysin receptor (ARNi) approved for HF treatment. On one hand, sacubitril determines a reduction in the preload and ventricular remodeling *via* its natriuretic, diuretic and vasodilator effects. On the other hand, valsartan expresses its benefic cardiovascular effects *via* selective blockade of AT I receptor and subsequent AT II inhibition. As a result, sodium and water retention and cardiorenal fibrosis are repressed (67).

Nevertheless, isolated utilization of neprilysin inhibitors enhanced plasma levels of AT I and AT II, proving the need of concomitant use of an angiotensin receptor blocker (ARBs) in order to achieve cardiovascular and renal benefic effects, and also the superiority of the combination in the treatment of HF patients (67, 68).

### NT-Pro ANP (Pro-atrial Natriuretic Peptide)

Its most important roles are natriuresis, diuresis, vasodilatation, inhibition of the renin-angiotensin-aldosterone system and blockage of the sympathetic nervous system (69, 70). As a general review, NPs act on three types of guanylyl cyclase receptors (NPR): NPR-A, NPR-B, NPR-C (69). Many factors can stimulate the secretion of ANP: increased intravascular volume, increased wall tension, chronic hypoxia, increased osmolarity (69). Given its sensitivity, we can use it to differentiate non-cardiac dyspnea from the cardiac one. We can consider, that it is a useful biomarker to diagnose HF (both chronic and acute) especially in outpatients with type 2 diabetes (69, 71), being also useful in predicting mortality, onset of atrial fibrillation and renal regression (69). Evidence suggest that its elevated plasma levels could also indicate a higher risk of major cardiovascular events and mortality, organ dysfunction and sepsis (70). The release of ANP depends on many factors, such as catecholamines, endothelins, arginine vasopressin and thyroid hormones (69).

### Corin

Corin represents a pro-natriuretic peptide convertase that together with NPs, plays an important role in the compensatory mechanism of maintaining fluid homeostasis in HF. Nonetheless, this compensatory mechanism seems to be limited since reduced plasma concentration of corin coupled with augmented plasma levels of NPs due to enzymatic downregulation (72), were observed in several studies amongst HF patients (26, 73, 74). For instance, levels of uncleaved pro-atrial natriuretic peptide (pro-ANP) were significantly (*p* = 0.01) elevated in HF patients and pre-clinical models, contributed to acute decompensations due to fluid disbalance (73, 74). Furthermore, Tripathi et al. (73) showed that on a genetic level, NPs transcripts increase in the last two stages of HF, while their plasma concentrations increased only in terminal HF (stage D). In contrast, cardiac corin transcripts begun to decline at early-stage B of HF (when patient do not exert symptoms of congestion), and remained low in evolution to stages C and D. To support this finding, another study exposed that experimental corin transcript restoration, significantly attenuates HF progression, in both HF and dilatative patients (75). The puzzling aspect in this last study is represented by the fact that corin, even in its catalytically inactive state, decreased congestion, thus proving that cardiac protective effects of corin do not necessarily require its protease status. Ultimately, corin evaluation in HF patients could stand as a marker of “disease progression” on a molecular level when other biomarkers such as NPs are not yet altered. This provides new insights in terms of early therapeutical intervention to prevent disease progression and onset of clinical symptoms. In addition, very low corin plasma levels combined with impaired pro-ANP cleavage and subsequently, very high levels of NPs (74), were found to be inversely corelated with the accumulation of lung edema and with the degree of decompensation.

### Biomarkers of Endopeptidase Activation

**Neprilysin**, a metalloprotease enzyme, is responsible for the degradation of the NPs, AT I and bradykinin, thus becoming a potential target for the treatment of congestive HF. Therefore, its inhibition was proposed as an adjuvant in diuretic therapy due to the rise in plasma concentrations of NPs with increased natriuresis, diuresis, and vasorelaxation (43). In this matter, following renal denervation, the beneficial effect was attributed to a significant inhibition of neprilysin in the kidneys due to its autocrine effects that exert cardioprotective actions and also combat congestion (9).

### Biomarkers of Inflamations, Fibrosis and Proteases

**Galectin-3** is a complex biomarker with pleiotropic physiological functions and documented pro-fibrotic activity myofibroblast proliferation that leads to ventricular remodeling and cardiac myocyte stress (76, 77). Studies on experimental animals proved that upregulation of galectin-3 was prone to exacerbate HF (77). Its role in clinical congestion and fluid disbalance may be attributable to its involvement in the inflammatory cascade following cardiac injury. As a matter of fact, genetic knockout of galectin-3 showed a reduction in cardiac inflammation and a slower disease progression thanks to a resistance of the left ventricle at pressure and volume overload (76, 78). Additionally, an extensive metanalysis including 7,057 patients with acute HF, presented elevated serum levels of galectin-3, which correlated with increased risk of all-cause mortality and cardiovascular mortality (79). In terms of prognostic value, it appears that galectin-3 has a superior power in predicting 60-day mortality as compared to the standard biomarker, NT-proBNP (78, 80). Surely, most recent findings agree that this novel biological molecule could be of great use in identifying patients at high risk of forthcoming events.

#### Soluble Suppression Tumorigenesis 2 (*sST2*)

Mechanical strain on cardiac fibroblasts and cardiomyocytes greatly induces the *ST2* gene, according to genomic research. A membrane-bound receptor (*ST2L*) as well as a soluble version of *ST2* are produced by this gene (*sST2*) (81). In instances of cardiac stress such as high blood pressure and myocardial hypertrophy (82) levels of *sST2* are significantly elevated. Furthermore, when associated with other biomarkers of myocardial injury such as high-sensitive Troponin (hsTn) and NT-proBNP, in unstable patients with acute HF, *sST2* increased the risk of death with a hazard ratio (HR) of 2.64 (66). The precise mechanism of *sST2* in HF remodeling and decompensation, however, is unknown, but it was established that its levels are elevated both in HFpEF and in HFrEF (83). Acute and chronic HF are also responsible for higher levels of *sST2* and it has been linked to poor prognosis and unfavorable LV remodeling (84). As for therapeutical implications, Gaggin et al. showed that *sST2* levels are specifically identified various strata of risk based on subsequent achievement of beta-blocker dosing, expanding the observation that *sST2* values are changed by beta-blocker dosing. Our findings open the possibility of prospectively investigating the use of *sST2*-guided beta-blocker treatment in HFrEF, since researchers in the field of chronic HF are currently tentatively examining the potential for biomarker-guided HF care (85).

#### Cardiac Bridging Integrator 1 (cBIN1)

A recent study exposed that cBIN1, a key mechanism in maintaining normal heart contraction, relaxation, and cardiomyocyte health, expresses various alterations in HF. Moreover, gene therapy with exogenous cBIN1 rescued cardiac lusitropic and inotropic properties, proving not only its great therapeutic potential in failing hearts (86), but also serving as a biomarker of cardiomyocyte remodeling (87).

Furthermore, Food and Drug Administration included Ischemia-Modified Albumin, electrocardiogram and the cardiac Troponin I as ischemia markers for the diagnosis of acute coronary syndromes (ACS), cumulating altogether a 95% sensitivity. Another biomarker of myocardial necrosis, Heart-type Fatty Acid-Binding Protein was proposed as an extra tool for the diagnosis of ACS, expressing only additive diagnostic value (66). However, its prognostic value for myocardial damage was established due to the fact that it proved to be the earliest marker for ACS confirmation (88).

Cathepsins are lysosomal proteases contributing to autophagic degradation of cellular substrates. They are composed of 11 members (cathepsin B, C, F, H, K, L, O, S, V, X, and W). Various findings support the notion that cathepsins could participate in cardiac remodeling by mediating extracellular matrix degradation. Cathepsin S and K were found increased in pressure overload-induced myocardium of patients with HF (89). Cysplatin-C is a potent cysteine protease inhibitor, that plays an essential role in vascular pathophysiology. It is an inhibitor of cathepsin S and K. The activity of cathepsin S is tightly regulated by its endogenous inhibitor, cystatin C, which also has a role in antigen presentation. Cathepsin S is a lysosomal enzyme that belongs to the papain-like protease of cysteine proteases, playing an active role in blood vessels permeability and angiogenesis. In normal situation, kidneys excrete Cysplatin-S. Any kind of kidney disease (glomerulopathy, tubulopathy, vasculopathy, interstitial disease) is first observed by high concentration of Cysplatin-C (90).

There was demonstrated, that Cysplatin-C has a major prognostic value in HFrEF. Cysplatin-C is a strong predictor of events in stable chronic HF, independent of traditional risk factors and BNP. Moreover, serum Cysplatin-C levels are correlated with glomerular filtration rate, so it can be useful in detecting kidney diseases. Recent studies demonstrated a direct correlation between high levels of Cysplatin-C and hospitalization rate and mortality. We can affirm, that Cysplatin-C could be a double predictor for acute/chronic kidney diseases and initial phase of HF (88).

**Figure 2:**
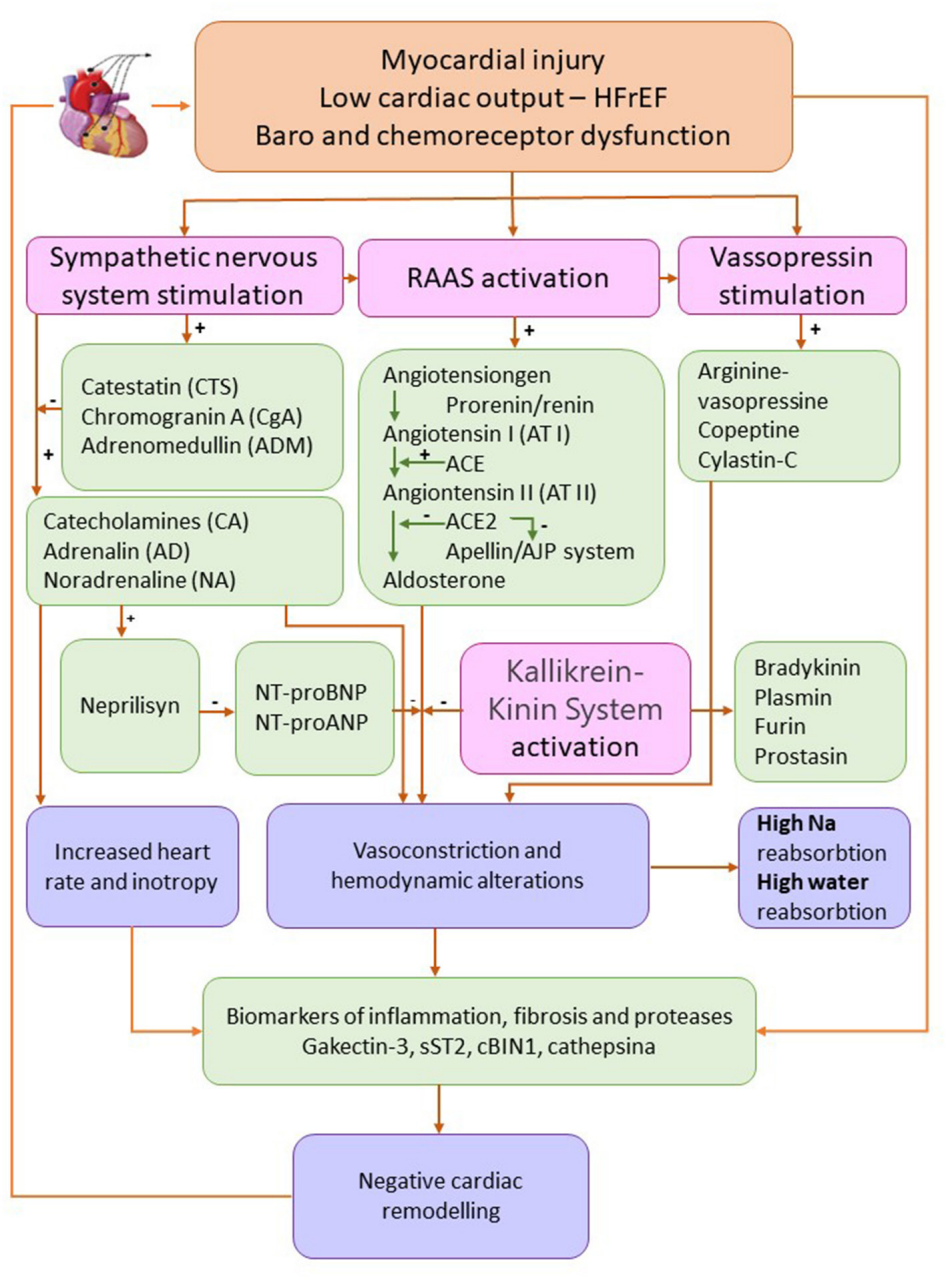
Biomarkers of different physiopathological pathways responsible for volume overload and edema heart failure with reduced ejection fraction. Adapted from Ibrahim et al. (84) and Oikonomou et al. (91) (HFrEF, heart failure with reduced ejection fraction; Na, sodium; RAAS, renin-angiotensin-aldosterone system).

Biomarkers of different physiopathological pathways responsible for volume overload and edema HFrEF are synthetized in Figure 2.

### Strengths and Limitations of the Review

This review summarizes the current data from the specialty literature regarding biomarkers that play a significant role in the pathogenesis of edema in HFrEF. Our work is meant to help clinicians working in cardiovascular medicine, express new targeted approaches in terms of pathophysiology, diagnosis, research clinical trials and new drugs development to reduce the morbidity and mortality in HF patients.

Several biomarkers have been studied in only few, limited scientific papers, underlying the need for continuous and rigorous further research. Hence, the lack of metanalysis focusing on their prognostic value as multimarker strategy in HF patients, encompasses our study limitation.

The biomarkers with clinical implications for edema in HFrEF are synthetized in Table 1.

**Table 1:** Biomarkers with clinical implications for edema in HFrEF.

**Biomarkers of sympathetic system activation**	**The role in HFrEF**	**Cellular mechanism**	**Cut-off values as diagnosis biomarkers**	**Role of prognosis biomarkers**	**Therapeutical consequences**
Catecholamines (CA) Adrenalin Noradrenalin (NA) (25)	Increase heart rate Favors vasoconstriction and sodium and water retention	Under stressful conditions, the CA are released from brainstem catecholaminergic neurons, spinal cord efferent nerves and adrenal medulla	Plasma circulating NA are higher in Stage B HF	CA are strongly linked to systolic dysfunction progression and to interstitial fluid overload	**Beta-blockers**
Catestatin Chromogranin A Precursor (CgA) (28, 29, 31)	Downregulation of CA release, providing cardioprotective effects	Produced by nerve fibers and neuroendocrine tissues	Increased circulating plasma levels in HF	Powerful predictive marker of mortality	Loop diuretics Beta-blockers
Adrenomedullin (ADM) (34, 35)	Vasodilation, anti-inflammation, tissue repair and organ protection	ADM secreted mainly by the gastrointestinal cells	Levels of plasma ADM are markedly increased in HF	Higher levels of plasma ADM are associated with greater risk of readmission due to decompensated HF	Loop diuretics
Prorenin/Renin (26)	Aggravates sodium and water reabsorption in the kidney	They are released from the juxtaglomerular apparatus in the RAAS cascade	Higher levels of plasma renin activity	Higher concentrations are associated with the onset of Stage C HF	Renin inhibitor (Aliskiren) delays progression of HF from stage B to stage C, and reduces edema
Aldosterone (8, 26, 42, 43)	Salt and water retention	Increase the apical permeability of the collecting ducts to sodium, modulating the activity of ENaC	Levels of plasma Aldosterone are markedly increased in HF		Mineralocorticoid receptor antagonists
ACE2 (54, 56)	Cardiovascular protection and inhibitor of Ang II	Degradation of Ang II	Higher concentration in HF	Biomarker of poor prognosis in HFrEF	
Apelin (52, 53)	Inotropic, vasodilator and diuretic effect	Kinin path activation Vasopressin inhibition in the brain	Low levels in advanced HF	Low levels are associated with poor prognosis in HF	Targeted apelin medication for HF (under research)
Bradykinin (BK) (61, 62)	Reabsorption vasodilation and reduction of sympathetic overdrive	A decrease in ENaC opening probability, thus blocking sodium and water	Significantly elevated urinary concentrations in plasmin, furin and prostasin, aggravating edema		ACEi increase BK levels
Natriuretic peptides (18, 64)	Diuretic and natriuretic effects	Ang II antagonists and reduce aldosterone secretion	Increased circulating plasma levels in HF	Associated with clinical worsening of HF, frequent hospitalization and higher mortality	Earlier therapeutical interventions and better clinical outcomes
Corin (73–75)	Compensatory mechanism of maintaining fluid homeostasis in HF	Pro-natriuretic peptide convertase	Cardiac corin transcripts begun to decline at early-stage B of HF	Marker of disease progression in HF Low levels are associated with poor prognosis in HF Inversely corelated with HF decompensation	
Neprilysin (NP) (43)	Fluid overload and vasoconstriction	Degradation of the NP, AT I and bradykinin	Increased circulating plasma levels in HF		Adjuvant in diuretic therapy
NT-pro ANP (69–71)	Diuretic and natriuretic effects, inhibition of RAAS	Mid-regional pro-atrial natriuretic peptide	Increased circulating plasma levels in HF	It can predict mortality, onset of atrial fibrillation and renal failure. Higher levels are associated with the risk of major cardiovascular events	
Copeptin (57–59)	Salt and water retention	Similar mechanism with vasopressin	Increased circulating plasma levels in HF	Higher levels are associated with poor prognosis in HF	
Soluble suppression of tumorgenicity 2 (*sST2*) (81, 82, 84, 85)	*sST2* is an interleukin-1 receptor member which reflects LV remodeling and fibrosis	Cardiac remodeling	Higher levels of *sST2* concentrations in patients with systolic HF	Higher levels of *sST2* associate a greater risk of 1-year mortality for patients with HFpEF and HFrEF	Beta-blockers
Galectin-3 (76–78)	Ventricular remodeling	Pro-fibrotic activity myofibroblast proliferation that leads to ventricular remodeling and cardiac myocyte stress	Increased circulating plasma levels in HF	Superior power in predicting 60-day mortality as compared to the standard biomarker, NT-proBNP	Drugs with effect on cardiac remodeling (ARNi, Mineralocorticoid Receptor Antagonists, ACEi)

*HFrEF, Heart failure with reduced ejection fraction; Na, sodium; RAAS, renin-angiotensin-aldosterone system*.

## Conclusion

Water retention and edema in HFrEF is a consequence of the redistribution of cardiac output and neurohormonal activation involving mainly the sympathetic system, the renin-angiotensin aldosterone system as well as the systems of quinines, endothelin, neuropeptides, and arginine-vasopressin. The in-depth study of the activation of these systems in HFrEF allows the identification of new markers with a prognostic and therapeutic value. The quantification of these biomarkers plays an important role in the timely initiation of an appropriate therapy against water retention and edema and thus it helps to prevent rehospitalizations and impaired cardiac function since these are considered strong determinants of mortality in those patients. Among the new biomarkers studied with a negative prognostic role, we mention the increase of the serum values of: catestatin, chromogranin A, adrenomedullin, ACE2, copeptin, MR-proANP, galectin-3, *sST2* as well as the decrease in apelin. Furthermore, the determination of these system-activation-specific biomarkers with a pathophysiological role in water and sodium retention, allows both the adequate modulation of the medication doses recommended by the guidelines and the development of new therapeutic means.

## Author Contributions

RC, R-DL, and DB research the literature. RC, R-DL, MM, and DB studied and analyzed the articles. RC, R-DL, and MM wrote the paper. All authors contributed to the article and approved the submitted version.

## Funding

This work was granted by project PDI-PFE-CDI 2021, entitled Increasing the Performance of Scientific Research, Supporting Excellence in Medical Research and Innovation, PROGRES, no. 40PFE/30.12.2021 and by a grant of the Romanian Ministry of Education 492 and Research, CCCDI - UEFISCDI, project number PN III-P2-2.1-PED-2019-1057, within PNCDI III.

## Conflict of Interest

The authors declare that the research was conducted in the absence of any commercial or financial relationships that could be construed as a potential conflict of interest.

## Publisher's Note

All claims expressed in this article are solely those of the authors and do not necessarily represent those of their affiliated organizations, or those of the publisher, the editors and the reviewers. Any product that may be evaluated in this article, or claim that may be made by its manufacturer, is not guaranteed or endorsed by the publisher.
